# Landscape of Stress Response and Virulence Genes Among *Listeria monocytogenes* Strains

**DOI:** 10.3389/fmicb.2021.738470

**Published:** 2022-01-20

**Authors:** Brankica Z. Lakicevic, Heidy M. W. Den Besten, Daniela De Biase

**Affiliations:** ^1^Institute of Meat Hygiene and Technology, Belgrade, Serbia; ^2^Food Microbiology, Wageningen University and Research, Wageningen, Netherlands; ^3^Department of Medico-Surgical Sciences and Biotechnologies, Sapienza University of Rome, Latina, Italy

**Keywords:** *L. monocytogenes*, stress genes, genomic islands, diversity, lineages, clonal complexes, low pH, persistence

## Abstract

The pathogenic microorganism *Listeria monocytogenes* is ubiquitous and responsible for listeriosis, a disease with a high mortality rate in susceptible people. It can persist in different habitats, including the farm environment, the food production environments, and in foods. This pathogen can grow under challenging conditions, such as low pH, low temperatures, and high salt concentrations. However, *L. monocytogenes* has a high degree of strain divergence regarding virulence potential, environmental adaption, and stress response. This review seeks to provide the reader with an up-to-date overview of clonal and serotype-specific differences among *L. monocytogenes* strains. Emphasis on the genes and genomic islands responsible for virulence and resistance to environmental stresses is given to explain the complex adaptation among *L. monocytogenes* strains. Moreover, we highlight the use of advanced diagnostic technologies, such as whole-genome sequencing, to fine-tune quantitative microbiological risk assessment for better control of listeriosis.

## Introduction

*Listeria monocytogenes* is a Gram-positive, facultative anaerobe, non-spore-forming, and psychro- and salt-tolerant organism. It is also a facultative intracellular pathogen, both for humans and animals. In susceptible people, including immunocompromised persons, infants, pregnant women, and older people, it can cause clinical manifestations with high mortality rates (Desai et al., 2019; Kayode et al., 2019; Schlech, 2019). Cases of human listeriosis often can be traced back to food products contaminated during production, on which the microorganism grows to high numbers. Special concern is in particular given to ready-to-eat (RTE) products, such as salads, deli meat, or smoked salmon, because these are consumed without a further heating step (Leong et al., 2015; EFSA Panel on Biological Hazards [Efsa Biohaz Panel]et al., 2018). Given the ubiquitous distribution of this microorganism, its transmission into food-processing facilities occurs either *via* raw materials or *via* equipment and employees. Once introduced in the facilities, several factors have been suggested to contribute to the ability of a strain to establish long-lasting colonization (Holch et al., 2013; Leong et al., 2014; Lakicevic and Nastasijevic, 2017; Stoller et al., 2019). Some authors hypothesized that a particular feature that makes the control of *L. monocytogenes* difficult in the processing environment is its capacity to survive or even to grow in different stressful conditions and to form biofilms (Gandhi and Chikindas, 2007; Ferreira et al., 2014; Gahan and Hill, 2014; Ariza-Miguel et al., 2015). The fast ability of *L. monocytogenes* to colonize food-processing facilities and the formation of persisters of some *L. monocytogenes* strains in various niches along the food chain have been described (Berrang et al., 2010; Leong et al., 2014; Bolocan et al., 2016). This suggests that persister cells provide reservoirs for contamination, ultimately increasing the likelihood of infecting humans. An alternative hypothesis is that there are no strains of *L. monocytogenes* with unique attributes but hard-to-reach areas (also known as harborage sites) in food industry environments and equipment where *L. monocytogenes* can reside (Carpentier and Cerf, 2011). Furthermore, tolerance to sanitizers and disinfectants such as benzalkonium chloride (BC) was observed in *L. monocytogenes* isolates from food-processing environments. This tolerance may be attributed to subinhibitory concentrations of a disinfectant, which are caused by insufficient cleaning and improper sanitation, thus probably contributing to biofilm formation and leading to *Listeria* persistence (Martínez-Suárez et al., 2016). The same authors have hypothesized that these subinhibitory concentrations cause the expression of stress response genes leading to a reduction in cell permeability to these compounds. These genetic traits include resistance genes such as the *qacH* gene of transposon Tn*6188* and the resistance cassette *bcrABC* (Elhanafi et al., 2010; Müller et al., 2013, 2014; Zuber et al., 2019), described in *Persistence mechanisms*. Moreover, it is crucial to identify the interactions between stress response and virulence and to know how this microorganism survives, adapts to adverse conditions, and triggers genes involved in virulence or promoting persistence. This would help to explain the observed inverse correlation between strains with a higher prevalence of genes involved in BC tolerance, as well as other stress-related genes, amongst hypovirulent (i.e., low virulence) lineage II strains (Quereda et al., 2021). All this knowledge may contribute to the development of new intervention strategies for better control of the level of *L. monocytogenes* in the food chain.

## *Listeria Monocytogenes* Diversity and Heterogeneity of the Virulence Determinants

*L. monocytogenes* evolves slowly but has been characterized by a significant level of diversity (Ragon et al., 2008; Orsi et al., 2011). It can be grouped into four major evolutionary lineages indicated by the roman numbers from I to IV, by 14 lineage-related serotypes and more than 170 clonal complexes (CCs),^1^ geographically and temporally widespread, as defined by multilocus sequence typing, and whole-genome phylogenetic analysis (Doumith et al., 2004; Orsi et al., 2011; Haase et al., 2014; Doijad et al., 2015; Chen et al., 2016; Moura et al., 2016; Bergholz et al., 2018). It belongs to the genus that currently includes 26 recognized species, of which notably 20 have been described since 2009 (Graves et al., 2010; Leclercq et al., 2010, 2019; Bertsch et al., 2013; Lang Halter et al., 2013; den Bakker et al., 2014; Weller et al., 2015; Doijad et al., 2018; Nuñez-Montero et al., 2018; Quereda et al., 2020; Carlin et al., 2021). Lineage I of *L. monocytogenes* encompasses serotypes 1/2b, 3b, 4b, 4d, 4e, and 7, lineage II includes serotypes 1/2a, 1/2c, 3a, 3c, and 4h, lineage III comprises serotypes 4a, atypical 4b, and 4c, whereas lineage IV encompasses serotypes 4a and 4c (Seeliger and Hohne, 1979; Ragon et al., 2008; Maury et al., 2016; Painset et al., 2019; Yin et al., 2019).

Previous studies have found that some hypervirulent clones such as CC1, CC2, CC4, and CC6 (all of lineage I and predominant in Western countries) were strongly associated with listeriosis, whereas hypovirulent clones, including CC8, CC9, CC101, CC121, and CC204 (lineage II), were strongly associated with food product contamination but less with human infections, in part due to loss-of-function mutations in virulence genes (Fagerlund et al., 2016; Maury et al., 2016, 2019). In accordance with that, all CC2 isolates carried a full-length *inlA* gene (see later), whereas CC9 and CC121 presented a premature stop codon mutation in this gene that correlated with reduced virulence (Gelbíčová et al., 2015; Guidi et al., 2021). Maury et al. (2019) demonstrated that differences in product associations among clones might be attributed to adaptation differences between clones in distinct ecological niches and/or different food product contamination routes during processing. They showed that CC1 was more representative for dairy products, whereas hypovirulent clones, mainly CC9 and CC121, were strongly associated with meat and fish products and produced more biofilm in the presence of low BC concentrations. Hypervirulent strains of *L. monocytogenes* sequence type (ST) 6 have been associated with outbreaks, including an outbreak linked to frozen vegetables in five countries in Europe during 2015–2018, an outbreak associated with contaminated meat pâté in Switzerland during 2016, listeriosis outbreak that occurred in South Africa during 2017–2018 with a 27% mortality rate, and the largest outbreak of listeriosis in Germany linked to blood sausages in 2018–2019 (Althaus et al., 2017; EFSA and ECDC, 2018; Halbedel et al., 2020; Thomas et al., 2020). More recently, an outbreak of listeriosis was caused by the persistence of *L. monocytogenes* serotype 4b ST6 (lineage I) in a cheese-processing facility in Switzerland (Nüesch-Inderbinen et al., 2021).

To cause listeriosis in humans and animals, important genes must be present in *L. monocytogenes* genome and expressed under the appropriate conditions. The *inlAB* locus and the pathogenicity islands LIPI-1, LIPI-3, and LIPI-4 encode such key virulence factors (Gelbíčová et al., 2015; Maury et al., 2016; Quereda et al., 2018). In particular, *inlA* and *inlB* code for internalin A (InlA) and internalin B (InlB) that bind the host cell receptors E-cadherin and Met, respectively. The transcriptional regulator positive regulator factor A (PrfA) controls the expression of both *inlAB* and LIPI-1 (Quereda et al., 2018).

LIPI-1, present in all *L. monocytogenes*, is found between the genes *prs* and *orfX*, is 9-kb long, and consists of six genes, i.e., *prfA*, *plcA*, *hly*, *mpl*, *actA*, and *plcB* (Dussurget, 2008). Notably, *Listeria innocua*, a non-pathogenic *Listeria* species, lacks both LIPI-1 and *inlAB* genes; however, LIPI-1 and *inlA*, functional both *in vivo* and *in vitro*, are present in rare, natural, atypical *L. innocua* species (Johnson et al., 2004; Volokhov et al., 2007; den Bakker et al., 2010; Moreno et al., 2012; Moura et al., 2019). This suggested that *L. monocytogenes* and *L. innocua* likely evolved from a common ancestor where the virulence loci LIPI-1 and *inlAB* were both present (Volokhov et al., 2007). *L. innocua* FSL J1-023 is one such aberrant strain, described as a rare, natural, non-pathogenic, hemolytic-positive, rhamnose and xylose fermentation-negative strain; its genome sequence is the reference one for linking horizontal gene transfer and recombination as drivers in the evolution of *Listeria* pathogenicity (Johnson et al., 2004; Lakicevic et al., 2014). Notably, all *L. innocua* genomes lack other internalins (i.e., *inlCEFGHJKP*), and this suggested that, unlike *inlAB*, the former genes were not present in the common ancestor and acquired at a later stage by *L. monocytogenes* (Moura et al., 2019).

As for LIPI-2, this pathogenicity island was discovered in *Listeria ivanovii*, a species pathogenic for feedstocks, mostly ovines and bovines, and humans, though rarely (Guillet et al., 2010). Originally, it was described as a LIPI specific to *L. ivanovii.* It encompasses the *smcL* gene coding for sphingomyelinase (involved in phagosome disruption) and 10 genes coding for proteins of the internalin family (Domínguez-Bernal et al., 2006). However, recent studies showed that *L. monocytogenes* isolates, belonging to a new sub-lineage of the major lineage II with hypervirulent features (SL626/CC33, serovar 4 h), contain a truncated LIPI-2, i.e., carrying only *smcL* and two internalins genes, namely *i-inlF* and *i-inlE*, likely acquired by transposon-mediated horizontal gene transfer from *L. ivanovii* (Yin et al., 2019; Feng et al., 2020).

**FIGURE 1:**
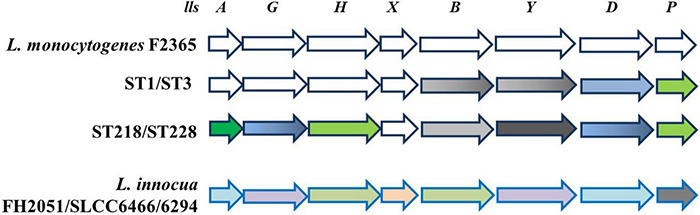
Schematic representation of LIPI-3 pathogenicity island. In *L. monocytogenes* reference strain F2365 and different STs, all LIPI-3 genes display SNPs, except *llsX*, which is strongly conserved. SNPs are shown as follows: 0—white; 1—gray; 2—green; 4—dark green; 10–17—blue; 18—black. When color is shaded, SNPs are present only in one of two ST indicated. Bottom line shows that LIPI-3 is also present in some strains of *L. innocua*, where sequence identity with *L. monocytogenes* reference strain F2365 ranges from 91% (*llsP*, gray) to 95% (*llsY* and *llsG*, violet), 97% (*llsX*, pink), 98% (*llsA* and *llsD*, light blue), and 99% (*llsH* and *llsB*, light green) (adapted from Clayton et al., 2014; Tavares et al., 2020).

LIPI-3 is an additional sub-lineage pathogenicity island encoding listeriolysin S (LLS), a bacteriocin. LLS (coded by *llsA*), a hemolytic toxin secreted by *L. monocytogenes*, is present only in a subset of isolates from lineage I epidemic strains that specifically secrete it in the gut (Quereda et al., 2017). Notably, Quereda et al. (2016) demonstrated that in an orally infected mouse model, *L. monocytogenes lls* mutants exhibited reduced bacterial load in the intestinal content at 6 h post-infection as compared with the wild-type strain, and the differences were also evident at 24 and 48 h post-infection and correlated with the reduced number of intracellular bacteria. Moreover, the same authors showed that the presence of *L. monocytogenes*-produced LLS in the intestine of the infected mice caused a significant decrease in the occurrence of different bacterial genera, such as *Alloprevotella*, *Allobaculum*, and *Streptococcus*. These results, for the first time, provided evidence that LLS plays an important role in the interaction with other species in the gut microbiota. In a study conducted by Matle et al. (2020), LIPI-3 was detected in isolates of which the majority were from lineage I, i.e., CC1, CC2, CC3, and CC228. Also, Roedel et al. (2019) found LIPI-3 in *L. monocytogenes* isolates belonging to CC1, CC3, CC4, CC6, and CC288. Tavares et al. (2020) described single-nucleotide polymorphism (SNP) of eight LIPI-3 genes (*llsAGHXBYDP*) of the four different STs (ST1, ST3, ST218, and ST288) compared with reference strain F2365 (lineage I). The authors revealed that LIPI-3 genes are well conserved in ST1 (serogroup IVb), whereas a number of SNPs were identified in ST3 (serogroup IIb), ST218 (serogroup IVb-v1), and ST288 (serogroup IIb). Within the LIPI-3 island, *llsX* is the only gene that is highly conserved among different LIPI-3-positive *L. monocytogenes* CC (Figure 1) and even in atypical hemolytic *L. innocua* (Figure 1). However, under acid stress conditions, only reference strain F2365 (4b) presented expression of *llsX* comparable with ST3 and ST288 (serogroup IIb, lineage I) strains, which points to acidic pH as an important environmental trigger (Clayton et al., 2014).

LIPI-4 pathogenic island is a cluster of six genes encoding a putative cellobiose family phosphotransferase system and shown to confer hypervirulence by enhancing invasion of the CNS and placenta (Maury et al., 2016). It is only found in some isolates of lineage I, i.e., CC2, CC4, and CC87 (Maury et al., 2016; Chen et al., 2018, 2020; Hilliard et al., 2018; Painset et al., 2019; Roedel et al., 2019; Matle et al., 2020; Zhang et al., 2020). It is known that CC2 has a worldwide distribution, whereas CC4 and CC87 are the most prevalent clones in France and China (Maury et al., 2016; Zhang et al., 2020). The latter CC was also responsible for two outbreaks in Guipúzcoa (Northern Spain) in 2013 and 2014 (Pérez-Trallero et al., 2014; Wang et al., 2019). In addition to LIPI-4, all CC87 strains contained a novel type II restriction–modification system with unknown significance (Wang et al., 2019).

Anaerobiosis represents an important trigger for virulence determinants because, in the gastrointestinal tract, the oxygen level gradually decreases and favors facultative anaerobic microorganisms such as *L. monocytogenes* (Horn and Bhunia, 2018). Indeed, Müller-Herbst et al. (2014) detected 28 non-essential genes that were upregulated only anaerobically, of which a subset were virulence-related genes, e.g., *inlB* and *Listeria* adhesion protein that is essential for full virulence (Burkholder et al., 2009). A complementary screening of an insertion mutant library of *L. monocytogenes* demonstrated that F_1_F_0_-ATPase (see *FoF1-ATPase, Glutamate decarboxylase, and Arginine and Agmatine deiminases*) is essential for anaerobic proliferation of *L. monocytogenes* (Müller-Herbst et al., 2014). Anaerobiosis also induced an acid tolerance response (Sewell et al., 2015), providing *L. monocytogenes* robustness to survive the stomach acidity and transit to the intestine. According to that, some CCs are more tolerant to low pH and colonize better the intestinal lumen (also known as host-associated hypervirulent clones) than those of hypovirulent CCs (Hingston et al., 2017; Maury et al., 2019).

All this information highlight that hypervirulent clones (CC1, CC2, CC4, and CC6) greatly connect with clinical cases, are predominantly found in lineage I, colonize better in the intestine lumen, cause listeriosis in a healthy host, and are strongly associated with dairy products. On the contrary, hypovirulent clones (e.g., CC9 and CC121), greatly connected with food and food-related isolates, are predominantly found in lineage II, causing listeriosis in immunocompromised patients, and are strongly associated with meat and fish products. All lineages of *L. monocytogenes* possess highly conserved LIPI-1 and *inlA/B* locus, which fits with the fact that the majority of hypovirulent clones and other lineage II isolates present truncated InlA, leading to virulence attenuation. Furthermore, comparative genomics analysis between hypo- and hypervirulent clones have uncovered the specific virulence clusters such as LIPI-3 (present approximately in 50% of lineage I strains) and LIPI-4. Regardless of these facts, the regulatory authorities consider all strains of *L. monocytogenes* to be equally pathogenic (Vázquez-Boland et al., 2020; Quereda et al., 2021), whereas the food safety risk attributed to different subgroups differ (Chen et al., 2006).

## *Listeria Monocytogenes* and Stress Resistance Genes

The resistance to environmental stresses such as acidic environment, nisin, bile acids, and high osmolarity is conferred to *L. monocytogenes* by stress resistance determinants located on the Stress Survival Islet 1 (SSI-1), whereas stress resistance determinants to alkaline and oxidative stresses are located on the SSI-2 (Hein et al., 2011; Guidi et al., 2021).

Generally, uncovering the resistance mechanisms to stressful conditions in food matrices and the environment is regarded as important to contribute to the development of novel and efficient measures to prevent contamination through the whole food chain continuum and control the growth of *L. monocytogenes* during food storage (Bucur et al., 2018).

### Stress Survival Islets 1 and 2

SSI-1, a five-gene stress survival islet [*lmo0444*, *lmo0445*, *lmo0446* (*pva*), *lmo0447* (*gadD1*), and *lmo0448* (*gadT1*)], has an equal distribution in human clinical isolates and in strains isolated from food and food-processing environments (Ryan et al., 2010). The presence of SSI-1, as well as the ability to form biofilms, correlated with the persistence of *L.monocytogenes* strains (Keeney et al., 2018). Notably, the strongest biofilms were formed by strains from serotype 1/2b, such as CC3 and CC5, the majority of which contained SSI-1, whereas serotype 4b (such as CC2 and CC6), most of which do not contain SSI-1, formed the weakest biofilms (Keeney et al., 2018).

According to Harter et al. (2017), SSI-2, consisting of the genes *lin0464* and *lin0465*, encoding a putative transcriptional regulator and an intracellular PfpI protease, respectively, is predominantly found in the hypovirulent strains of *L. monocytogenes* ST121. SSI-2 contributes to survival upon oxidative and alkaline stress conditions, thus potentially favoring *L. monocytogenes* adaptation and persistence in the food-processing environments. Notably, in addition to SSI-2, the genome of *L. monocytogenes* ST121 possesses plasmids and the transposon Tn*6188*, which were hypothesized to be responsible for supporting its survival in food-processing environments (Müller et al., 2014; Schmitz-Esser et al., 2015; Pasquali et al., 2018). SSI-2 occasionally is found in other *L. monocytogenes* strains, such as ST1033 (CC1, serotype 4b).

Interestingly, SSI-2-positive strains were detected in *L. monocytogenes* lineage I, lineage III, and also in *L. innocua* but with a slightly shorter islet harboring only 1,947 bp (Harter et al., 2017). Phylogenetic analysis indicated that *L. innocua* SSI-2 shares the highest similarity with those of *L. monocytogenes* strains ST13 and CC193 that belong to lineage II (i.e., all food isolates) (Harter et al., 2017).

### F_o_F_1_-ATPase, Glutamate Decarboxylase, and Arginine and Agmatine Deiminases

**FIGURE 2:**
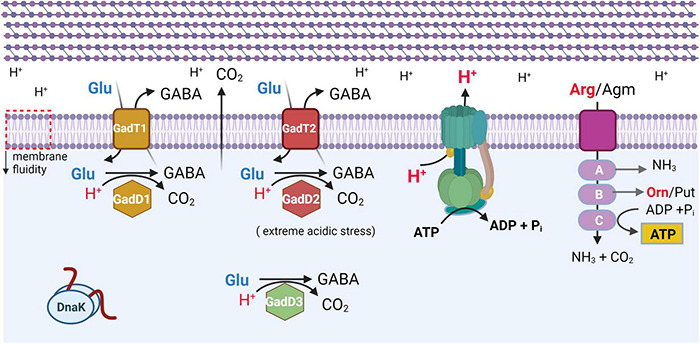
Schematic representation of most effective systems protecting *L. monocytogenes* from acidic stress. All systems are activated under mild acidic stress, with GadD2T2 system mostly effective under extreme acidic stress (adapted from Lund et al., 2020). F_0_F_1_-ATPase is shown in blue-green. ArgD/AguD transporter is shown in purple. A, ArcA (arginine), AguA1 (agmatine) deiminase; B, ArcB/AguB carbamoyl-transferase; C, ArcC/AguC carbamate kinase. GadT1 and GadT2 refer to glutamate (Glu)/GABA antiporters. GadD1, GadD2, and GadD3 are glutamate decarboxylase isoforms. Orn, ornithine. Put, putrescine. DnaK, chaperone. All membrane proteins are localized in lipid bilayer of plasma membrane, which also undergoes a decrease in membrane fluidity (downward pointing arrow, on leftmost side). Multilayered peptidoglycan of cell wall is schematically represented above plasma membrane. Created with BioRender.com.

Notably, SSI-1 contains *gadD1* and *gadT1*, which are among the genes protecting from acid stress. Indeed, the ability to tolerate a low pH environment is an important feature of *L. monocytogenes* because it allows survival in acidic environments encountered in the gastrointestinal tract of the host, in the macrophage phagosome, and in natural and food-processing environments (Gahan and Hill, 2014; Lund et al., 2014; Arcari et al., 2020; Lund et al., 2020). *L. monocytogenes* harbors membrane-associated systems and intracellular systems to resist acidic environments and to control intracellular pH. Several mechanisms, schematically depicted in Figure 2, are known to maintain intracellular pH (pH_i_) to values compatible with *L. monocytogenes* vitality. These include the F_0_F_1_-ATPase, the glutamate decarboxylase (GAD) system, and the arginine and agmatine deiminases (ADI and AgDI, respectively) (Cotter et al., 2000; Feehily et al., 2014; Cheng et al., 2017). The F_0_F_1_-ATPase, a multi-subunit enzyme system, is involved in the acid tolerance response initiation upon mild acidic pH stress. The GAD system, on the other hand, can affect survival under mild acid stress but also under harsher acidic conditions (Karatzas et al., 2012). Notably, the GAD system is also activated under low oxygen availability, typically encountered by *L. monocytogenes* when exposed to food packaging atmosphere (Francis et al., 2007; Sewell et al., 2015). The system consists of three homologous glutamate decarboxylases, namely GadD1, GadD2, and GadD3, and of the cognate glutamate/GABA antiporters GadT1 and GadT2 (Figure 2). The decarboxylases and the antiporters are encoded by the relevant genes at three distinct genetic loci. Four genes are organized in the following operons: *gadD1T1* and *gadT2D2*, whereas the *gadD3* gene, the fifth gene, is an independent unit (Cotter et al., 2005). In particular, the *gadD1T1* operon enhances growth under mildly acidic conditions (see also *Stress survival islets 1 and 2*), whereas *gadT2D2* plays an important role in conferring survival under extremely acidic conditions (Cotter et al., 2001, 2005). The *gadD3* gene, positively regulated by the stationary-phase sigma factor σ^B^, in addition to being part of the GAD system, was shown to be involved in nisin resistance (Begley et al., 2010). According to Chen et al. (2012), *gadD2* and *gadD3* are present in all *L. monocytogenes* strains, whereas *gadD1* is present in only 36.6% of the strains, including all those belonging to serovar 1/2c, and 68.5% of the strains of serovar 1/2a. Notably, only a small fraction of the strains of *L. monocytogenes* serovar 1/2b and lineage III strains (including J2-071 and HCC23) possess the *gadD1* gene. Furthermore, the *gadD1T1* operon is absent in most serotype 4 *L. monocytogenes* clinical strains (Cotter et al., 2005).

Paudyal et al. (2020) have shown that casamino acids, peptone, and tryptone are major GAD system activators resulting in upregulation of the transcription of *gadD2*. Furthermore, Paudyal et al. (2018) demonstrated that maleic acid inhibits the GAD of *L. monocytogenes* significantly, enhancing its sensitivity to acidic conditions, and thus, together with the ability to remove biofilms, maleic acid has been proposed to make a good candidate for disinfection regimes.

Moreover, Boura et al. (2020) recently showed a secondary novel role of the GAD system in protection against oxidative stress. The authors hypothesized that under oxidative stress GABA, instead of being exported by the transporter (Figure 2), is transferred to the GABA shunt enzymes that provide NADPH to contrast oxidative stress and allow to bypass two missing steps in the TCA cycle. They also suggested that this knowledge could find application in food hurdle technology to eliminate *L. monocytogenes*.

In addition to the GAD system, also the ADI system can be activated in response to low pH. This latter system consists of three enzymatic activities, namely arginine deiminase, ornithine carbamoyl-transferase, and carbamate kinase, encoded by the *arcA*, *arcB*, and *arcC* genes, respectively (Cheng et al., 2017). Through these enzymatic activities, arginine is converted to ornithine, CO_2_, and ammonia, with concomitant production of adenosine triphosphate (Figure 2). It was previously reported that this gene cluster is present in lineage I and lineage II but absent from lineage III and non-pathogenic *L. innocua* and *Listeria welshimeri* (Ryan et al., 2009). However, Deng et al. (2010) showed that the ADI gene cluster is also highly conserved in lineage IIIB.

The AgDI pathway is a less known system of acid stress tolerance in *L. monocytogenes*. AgDI converts agmatine into putrescine, ammonia, and CO_2_ and produces adenosine triphosphate (Chen J. et al., 2011; Soares and Knuckley, 2016). Cheng et al. (2013) found that *L. monocytogenes* harbors two putative AgDIs (*aguA1* and *aguA2*), but only AguA1 functionally participates in the AgDI pathway and mediates acid tolerance in *L. monocytogenes*.

Also, the thiamine uptake system, encoded by the *thiT* gene (formerly *lmo1429*), was shown to be required for full acid tolerance in *L. monocytogenes* (Madeo et al., 2012). According to the authors’ findings, a *thiT* mutant strain resulted in significantly higher acid sensitivity than the control strain. It was suggested that the acid sensitivity is due to the lack of thiamine that does not allow the reaction of acetolactate synthase to occur, and therefore, acetoin synthesis, which involves proton consumption, is impaired (Madeo et al., 2012).

When different *L. monocytogenes* strains are exposed to acidic environments, the acid tolerance displayed varies significantly, and this might contribute to the observed strains’ differences in robustness and pathogenicity (Conte et al., 2000). The virulent reference strains *L. monocytogenes* EGD-e (1/2a, lineage II), 850658 (4a, lineage III), and 10403S (1/2a, lineage II) were more resistant to acidic stress than the avirulent M7 (4a, lineage III), which showed a defect in maintaining pH_i_ homeostasis (Cheng et al., 2015). Despite the observations mentioned, EGD-e cannot export GABA and relies exclusively on GadD3, whereas strain 10403S relies upon GadD2 (Feehily et al., 2014). This suggested that the GAD system in the commonly used reference strains originated from separate lines of evolution. Strain-specific patterns of acid resistance are also recognizable in other datasets (Ramalheira et al., 2010).

### σ^B^ Regulon

The sigma factor of RNA polymerase, responsive to general stress, namely σ^B^, plays an important role in *L. monocytogenes*, both in the adaptation to different stress conditions and in virulence. As shown by many studies conducted using the lineage II reference strains (mainly the 10403S as mentioned earlier and EGD-e), σ^B^ regulates approximately 300 genes important for virulence and responses to stresses (Severino et al., 2007; Hain et al., 2008; Raengpradub et al., 2008). Also, the literature review revealed that the σ^B^ regulon played a significant role in the resistance of *L. monocytogenes* strains belonging to lineages I, II, and IIIB and a limited role in the resistance of the *L. monocytogenes* lineage IIIA strain to acid and oxidative stresses (Oliver et al., 2010; Liu et al., 2019). Similarly, σ^B^ plays a significant role in resistance to acid and salt stresses also in *L. innocua* (Raengpradub et al., 2008).

A systematic review of the σ^B^ regulon in *L. monocytogenes* identifies several regulon members that include genes involved, or putatively involved, in stress response: osmotic (18 genes), oxidative (14 genes), acid (12 genes), antibiotic (6 genes), bile (3 genes), and others (24 genes) (Liu et al., 2019). However, σ^B^ is not the only alternative sigma factor that has been shown to play a role in the stress tolerance of *L. monocytogenes.* Other alternative sigma factors, including σ*^C^* (previously implicated in nisin response), σ*^H^*, and σ*^L^* (RpoN), also regulate transcription of genes important for virulence and response to various stress and growth conditions (Glaser et al., 2001; Chaturongakul et al., 2011). Some of the genes needed in the stress responses of *L. monocytogenes* are regulated by more than one sigma factor. Overlaps have been reported between σ^B^ and σ*^H^*, σ^B^ and σ*^L^*, and σ*^C^* and σ^B^ regulons (Chaturongakul et al., 2011). Notably, the regulation of SSI-2 (see *Stress survival islets 1 and 2*) is independent of the σ^B^, but σ*^H^* or σ*^L^* could be involved (Harter et al., 2017). Interestingly, of the 51 genes classified in the “virulence” group of the σ^B^ regulon, 23 were also classified in the “stress response” group and 4 in the “metabolism” group (Liu et al., 2019). The genes that belonged both to the “virulence” and “stress response” groups included those required for survival and multiplication under host-imposed stress conditions such as acidic pH and bile acids in the gastrointestinal tract of the host and those contrasting oxidative stress in the mammalian host phagosome. These observations reinforced the hypothesis that stress response and virulence are strongly associated. Among the 24 genes of the σ^B^ regulon that belong only to the “virulence” group, there is the gene coding for the PrfA, located in LIPI-1 (see *L. monocytogenes diversity and heterogeneity of the virulence determinants*), which can be transcribed from multiple promoters, regulated by σ^B^, σ*^A^*, and PrfA itself (De Las Heras et al., 2011).

### Resistance to Nisin and Envelope Remodeling

Natural antimicrobials, such as nisin (which belongs to the class I bacteriocins), sakacins, pediocin PA-1, plantaricin BM-1, and leucocin A (which belong to the class II bacteriocins), can be used to control *L. monocytogenes* on RTE foods (Ferreira and Lund, 1996; Nilsson et al., 1997; Franklin et al., 2004; Trinetta et al., 2010; Woraprayote et al., 2013; Ortiz et al., 2014; Balay et al., 2017; Xie et al., 2018). However, nisin is the only bacteriocin approved as a preserving additive in food. It should be highlighted that RTE foods add up further stress to *L. monocytogenes* when these foods are exposed to cold stress, organic acid stress, and osmotic stress, and some of these environmental stresses may affect bacteriocin resistance, e.g., nisin resistance increases if *L. monocytogenes* is preexposed to acid stress (van Schaik et al., 1999; Bonnet and Montville, 2005).

VirR, the response regulator of the VirRS two-component system, with a role in defense against cell envelope stress (Mandin et al., 2005), is directly controlling nisin resistance. In this specific case, instead of the receptor histidine kinase VirS, an ABC-transporter encoded by *virAB* seems to be responsible for sensing the stressor (Grubaugh et al., 2018). VirR mediates nisin and other cell envelope stress resistance by regulating the *dltABCD* operon (Kang et al., 2015), which modifies lipoteichoic acids (Abachin et al., 2002). In addition to VirR, the two-component systems LiaRS and LisRK were shown to be involved in resistance to nisin (Cotter et al., 2002; Collins et al., 2012; Bergholz et al., 2013; Nikparvar et al., 2021). In particular, these two-component systems regulate the expression of *anrAB*, *dltABCD*, *lmo2229*, *mprF*, and *telA* (Abachin et al., 2002; Gravesen et al., 2004; Thedieck et al., 2006; Collins et al., 2010a,b, 2012). With the exception of the latter, all the listed genes have an assigned role in the biosynthesis/metabolism of components of the membrane and the cell wall. Using a laboratory cheese model, temperature and pH were shown to be among the environmental conditions that affected the sensitivity of *L. monocytogenes* to nisin, the efficacy of which was stronger when the cheese was stored at low temperatures and prepared at pH close to neutrality (i.e., pH 6 and 6.5), due to the activity of *dltA* and *mprF* (Henderson et al., 2020).

The resistance to nisin varies among the various lineages, with lineage II strains being more tolerant than lineage I strains. These differences extended to serotype and CC levels. For example, serotypes 1/2a and 1/2c were more tolerant than serotype 1/2b, whereas serotype 4b showed the least tolerance to nisin, as also supported by studies showing that serotype 1/2a and 4b strains were more tolerant and sensitive to nisin, respectively (Buncic et al., 2001; Katla et al., 2003; Szendy et al., 2019; Wambui et al., 2020). Comparing clonal types, Wambui et al. (2020) concluded that CC7 (lineage II, 1/2a) strains displayed the highest nisin resistance, whereas CC2 (lineage I, 4b) and CC3 (lineage I, 1/2b) strains showed the lowest nisin resistance levels, similar to previous observations (Malekmohammadi et al., 2017). In addition, among CCs belonging to lineage II serotype 1/2a, CC155 was the most nisin tolerant, whereas CC14, CC199, and CC403 (lineage II, serotype 1/2a) had increased nisin sensitivity. Differences in the ability to respond to stress among the *L. monocytogenes* genotypes are probably linked to differences in the expression of proteins associated with the membrane, such as the penicillin-binding proteins coded by the *lmo0441*, *lmo0540*, *lmo1892* genes, and *lmo2229* as mentioned earlier, as well as the gene coding for σ^B^, all of which were reported to be more expressed in lineage II than lineage I (Gravesen et al., 2004; Begley et al., 2006; Severino et al., 2007).

## Persistence Mechanisms

In food factories, sanitizer tolerance or resistance and an enhanced ability to form biofilms have been suggested as typical strain characteristics contributing to persistence (Norwood and Gilmour, 1999; Aase et al., 2000; Lundén et al., 2000, 2003; Borucki et al., 2003; Heir et al., 2004; Pan et al., 2006; Lourenço et al., 2009). However, the exact mechanisms behind persistence are not fully understood. Difficulties in eradicating *L. monocytogenes* contamination in food-processing settings may be conferred by the BC tolerance that some strains harbor and which provides an advantage for survival under stress and in food-processing settings, allowing the bacteria to persist in the environment (Mullapudi et al., 2008). A recent publication by Guérin et al. (2021) reported the capacity of *L. monocytogenes* strains to adapt to biocides, in particular ammonium quaternary compounds (commonly known as quats or QACs), and proposed the possible link between this adaptation and the selection of resistance regarding the fluoroquinolone antibiotic ciprofloxacin. This link was investigated also by others (Martínez-Suárez et al., 2016; Kode et al., 2021). The current hypothesis is that dilution in the environment and biodegradation give rise to QAC concentration gradients, which means that microorganisms (including *L. monocytogenes*) become frequently exposed to sub-inhibitory concentrations of QACs (Martínez-Suárez et al., 2016). Indeed, Møretrø et al. (2017) measured and found residues of QACs after sanitation in meat- and salmon-processing plants in Norway and suggested that this may result in a growth advantage for *L. monocytogenes* harboring the QAC resistance genes (i.e., *qacH* and *bcrABC*; see later). Therefore, the low-level resistance to QACs in *L. monocytogenes* has been proposed to be a contributing factor to its environmental adaptation and persistence. This may explain why the minimal inhibitory concentration of QAC tolerant strains way below user concentrations of QAC may still be of practical relevance. More studies are needed to confirm this hypothesis. Another study conducted by Castro et al. (2021) demonstrated that mobile genetic elements (MGEs) support the persistence of *L. monocytogenes* on dairy farms and may be spread through the food industry. It is believed that MGEs are pivotal in increasing the antimicrobial resistance of *L. monocytogenes* strains. MGEs can be exchanged between *Listeria* and other species leading to the creation of novel resistance phenotypes (Matereke and Okoh, 2020; Castro et al., 2021).

Several BC tolerance determinants have been identified in *L. monocytogenes*, including BC efflux pumps *qacH* (Tn*6188*), *bcrABC*, and *emrE*, which are located on MGEs and mostly present in lineage II isolates, i.e., CC9, CC13, CC14, CC31, and CC121 (Dutta et al., 2013; Müller et al., 2013, 2014; Ebner et al., 2015; Kovacevic et al., 2015; Maury et al., 2016; Ortiz et al., 2016; Zuber et al., 2019). Additional BC tolerance genes have been identified, such as *emrC*, identified on a plasmid in some ST6 isolates, *qacA* and *qacC*, which are both located on plasmids, and multidrug resistance *Listeria mdrL* (*lmo1409*), negatively regulated by *ladR* (*lmo1408*) (Xu et al., 2014; Kremer et al., 2017; Jiang et al., 2019). Literature data on the presence of resistance genes are sometimes contradictory and not always related to all strains of a particular *L. monocytogenes* CC. For example, Hurley et al. (2019) and Chen et al. (2020) did not find *emrC* in ST6, as previously described by Kremer et al. (2017) in ST6.

Several very recent investigations showed that gene *qacH* can occasionally be present in some different CC primarily of serotype 1/2a (CC8, CC20, CC31, CC101, and CC121) but also in serotypes 4b (CC2) and 1/2c (CC9) (Ebner et al., 2015; Meier et al., 2017; Horlbog et al., 2018; Roedel et al., 2019; Stoller et al., 2019; Zuber et al., 2019; Wieczorek et al., 2020; Gelbicova et al., 2021; Guidi et al., 2021; Palaiodimou et al., 2021; Pérez-Baltar et al., 2021). In general, Cherifi et al. (2018) recommended that only one genetic determinant should not be taken into consideration when strain persistence is investigated.

The *bcrABC* cassette was first described by Elhanafi et al. (2010) and isolated from *L. monocytogenes* strains associated with the 1998–1999 listeriosis outbreak in the United States caused by hotdog contamination. It consists of a TetR family transcriptional regulator (*bcrA*) and two small multidrug resistance genes (*bcrB* and *bcrC*). This cassette is located in the pLM80 plasmid, but a chromosomal location was also reported. The occurrence of the *bcrABC* cassette in non-pathogenic species of *Listeria*, such as *L. innocua* and *L. welshimeri*, suggests that these species may be the reservoirs of BC and other resistance determinants that are transferred to *L. monocytogenes* by conjugation (Katharios-Lanwermeyer et al., 2012). The *bcrABC* cassette was significantly associated with *L. monocytogenes* isolates belonging to CC321, CC155, CC204, and CC199 but can be present in other CCs such as ST14, CC288, ST9, ST121, CC5, *L. welshimeri*, and *L. innocua* (Meier et al., 2017; Møretrø et al., 2017; Pasquali et al., 2018; Chen et al., 2020; Naditz, 2020; Cooper et al., 2021; Gelbicova et al., 2021; Palaiodimou et al., 2021).

Another putative efflux pump gene responsible for increased tolerance to QACs is the *emrE* gene. This gene is located on a mobile genomic island LGI1 and was found in one clone (CC8) that includes strains implicated in the 2008 deli meat outbreak in Canada (Kovacevic et al., 2015). Meier et al. (2017) concluded that the *emrE* is associated with a serotype 1/2a (CC8) and seems to be limited to sublineage 8 strains. The expression of *emrE* was found to be upregulated in the presence of BC, demonstrating that *emrE*-harboring strains are likely to adapt in food-processing environments better (Kovacevic et al., 2015).

The link between BC resistance and cadmium (Cd) resistance in *Listeria* spp. strains has been reported in several studies (Mullapudi et al., 2008; Ratani et al., 2012; Korsak and Szuplewska, 2016). In particular, in *L. monocytogenes* strains, five Cd resistance determinants (*cadAC* efflux systems) were identified (Chmielowska et al., 2021). The *cadA1* gene is located on the transposon Tn*5422* and often plasmid-borne, and predominates (as operon *cadA1C1*) in CC3, CC8, and CC121 (Lebrun et al., 1994; Gelbicova et al., 2021). Also, the *cadA2* gene is usually found on plasmids and is typically accompanied by the *bcrABC* cassette (Kuenne et al., 2010; Dutta et al., 2013). As for the genes *cadA3*, *cadA4* (also involved in biofilm formation), and *cadA5*, their location is typically on chromosomes, as part of integrative conjugative elements and genomic islands (LGI2 and LGI2-1), respectively (Kuenne et al., 2013; Lee et al., 2017; Parsons et al., 2017, 2019). Notably, LGI2, in addition to carrying *cadA4*, also carries a cassette for resistance to arsenic, which encompasses the *arsR1D2R2A2B1B2* operon and the upstream *arsA1D1* (Kuenne et al., 2013). The arsenic resistance is primarily associated with *L. monocytogenes* strains belonging to 4b serotype, particularly CC1, CC2, and CC4 hypervirulent clonal clones (Lee et al., 2017). On the contrary, Cd resistance typically is found in *L. monocytogenes* strains belonging to serotypes 1/2a and 1/2b, from food and food-processing environments (Mullapudi et al., 2008; Ratani et al., 2012). In addition, a specific association with lineages was found, namely *cadA1C1* cassette with lineage II and *cadA2C2* cassette with lineage I. On the other hand, strains containing both *cadA1* and *cadA2* were more frequent in lineage I than in lineage II (Mullapudi et al., 2010).

As said at the beginning of this section, the enhanced ability to form a biofilm that is hard to remove mechanically and less sensitive to sanitizers was proposed as a mechanism for persistence, given that the biofilm provides a clear advantage for surviving in food-processing or retail environments. However, other studies have not found a clear link between the biofilm-forming ability of some isolates and their persistence, and differences in the experimental setup and in the strains used have been ascribed as the reasons for the observed different results (Djordjevic et al., 2002; Holch et al., 2013; Kadam et al., 2013; Lee et al., 2019). Although some authors reported a correlation between lineages and biofilm-forming ability, with lineage II strains presenting higher levels of biofilm production, other results did not support these findings (Norwood and Gilmour, 2001; Djordjevic et al., 2002; Borucki et al., 2003; Di Bonaventura et al., 2008; Takahashi et al., 2009; Combrouse et al., 2013; Bai et al., 2021). Maury et al. (2019) found that hypovirulent genotypes, CC121 and CC9, were more efficient in biofilm production than hypervirulent clones (such as lineage I clones: CC1, CC2, CC4, and CC6) under sub-lethal concentrations of BC, implying that lineage II hypovirulent clones were associated with persistence features. Also, Pérez-Baltar et al. (2021) found that CC121 strains are strong biofilm formers, and some harbored the transposon Tn*6188*, related to increased tolerance to QACs. Interestingly, the *lmo0435* homolog biofilm-associated protein, BapL, putative peptidoglycan bound protein involved in biofilm formation, but not essential, is truncated in ST121 strains, which belong to CC121 (Jordan et al., 2008; Schmitz-Esser et al., 2015). Some authors suggested that CC8 strains possess a strong capacity for biofilm formation, which may support persistence within food production environments and subsequent contamination of foods (Verghese et al., 2011; Zuber et al., 2019). A study done in Canada by Upham et al. (2019) found that the formation of biofilms is associated with serotype 1/2a isolates in lineage II, as well as the presence of SSI-1. SSI-1, rare in clinical isolates, has been shown to be associated with a survival advantage in the environment, thus supporting the link between SSI-1 and persistence in *L. monocytogenes* (Hilliard et al., 2018). Furthermore, SSI-1 was strongly correlated with biofilm formation and a truncation (stop codon) in *inlA* (Franciosa et al., 2009; Keeney et al., 2018). More recent findings confirmed the influence of SSI-1 and a truncated *inlA* in increased biofilm levels in *L. monocytogenes* (Ciccio et al., 2019).

Regarding niche preference, reasons why the so-called persistent isolates are recurrently isolated in the same food-processing premises over long periods remain elusive. Persistent isolates could belong to specific STs particularly well adapted to the environmental conditions of the food manufacturing environment (Knudsen et al., 2017). It is, however, difficult to pinpoint adaptive traits directly correlated to persistence.

Notably, intra-genotype variation was observed in some CCs, suggesting that minor genetic variants within a genotype may impact biofilm phenotype (Lee et al., 2019; Zuber et al., 2019). Nevertheless, it should be pointed out that next to genetic determinants, biofilm formation is influenced by factors such as temperature, nutrient availability, and biofilm formation maturity (de Oliveira et al., 2010; Barbosa et al., 2013; Kadam et al., 2013). The limitation of most biofilm studies is that they have been done in monocultures, which may not be most relevant, as this bacterium is not alone in the food industry. Therefore, research on multispecies community might be more suitable to gain better insights into interactions among different species within the biofilms and the formation of the biofilm itself.

## Opportunities of Whole-Genome Sequencing for Quantitative Microbiological Risk Assessments

Whole-genome sequencing (WGS) of strains that have been isolated from different ecological niches has become more and more standard practice and demonstrates to be powerful in outbreak investigations at national and international levels (ECDC, 2019). These outbreak investigations rely on cross-sectorial cooperation between epidemiologists, microbiologists, and bioinformaticians to link clinical isolates to outbreak isolates. The availability of WGS data gives new opportunities to explain intraspecific variability and to find genetic biomarkers that predict microbial behavior. Multilocus sequence typing and whole-genome phylogenetic analyses demonstrated that pathogenic subtypes vary in their virulence and association with food (*L. monocytogenes diversity and heterogeneity of the virulence determinants*), and this intraspecific variability is relevant for risk assessments in general and, more specifically, also for quantitative microbiological risk assessments (QMRA). A QMRA is a structured and quantitative process for determining the risk associated with microbiological hazards in a food (CAC, 1999). The basic steps of a QMRA include hazard identification, exposure assessment, hazard characterization, and risk characterization, and this formalized approach has been adopted by regulators globally and is also used by industry. Various QMRA studies have been performed for different product/pathogen combinations (e.g., FDA/CFSAN and USDA/FSIS, 2003; Tirloni et al., 2018; EFSA, 2019) aiming to characterize and quantify the risk of a pathogen associated with a product (category). In these studies, the exposure assessment and hazard characterization steps of QMRAs are performed for a pathogenic species as a whole. The current advances in the field of omics technology give opportunities to make use of the greater understanding of intraspecific variability based on various recently published bioinformatics tools (Brul et al., 2012; Den Besten et al., 2018; Haddad et al., 2018; Rantsiou et al., 2018; Fritsch et al., 2019; Njage et al., 2020). Instead of considering all-hazard strains of a species as equally likely to cause disease or equally likely to survive the food chain, WGS data could give support to rank subtypes with respect to their virulence potential (Chen Y. et al., 2011; Collineau et al., 2019) or to groups subtypes with respect to their differences in robustness or fitness to reach the consumer stage (Den Besten et al., 2018). The QMRA input distributions can be tailored to each subgroup accordingly, making it possible to fine-tune the QMRA output. The studies of Chen Y. et al. (2011) and Fritsch et al. (2018) illustrate the potential to refine QMRA studies when considering pheno-genotype associations for specific properties of *L. monocytogenes*. The authors described the variability of *L. monocytogenes*’ growth characteristics more accurately using two different distributions for the minimum temperature of growth (T_min_). For risk characterization, three different groups of virulence were considered according to the CCs, making use of reported differences in clinical frequencies of different CCs (Maury et al., 2016, see also *L. monocytogenes diversity and heterogeneity of the virulence determinants*). The QMRA output showed that CCs that are contributing the most to consumer exposure were not those that contributed the most to listeriosis cases. Chen Y. et al. (2011) followed a similar approach for the hazard characterization step where they attributed different dose-response models for *L. monocytogenes* subtypes with genes encoding a full-length and a truncated InlA, respectively. These examples of fine-tuning a QMRA highlight the potential impact of implementing genomic data in QMRA. This field is still young and relies on high efforts to phenotypically characterize strain variability. Grouping of strains with shared characteristics is only possible when subgroups of strains have different phenotypes. This pushes the need to characterize various aspects of strains, such as fitness and stress robustness, because these details are needed to quantitatively describe intraspecific variability in the exposure assessment part of a QMRA. Also, it is important to note that routine collection of WGS data is more standardized across regulatory and public health agencies and more limited in surveillance by industry (Jagadeesan et al., 2019; Cohn et al., 2021), and this introduces a bias in isolate characterization, whereas representative WGS data from food and human isolates are critical to assess the likelihood of subtypes to cause disease. When these challenges are recognized and taken up, it will open avenues to make use of pheno-genotype associations in the next generation of QMRA and to incorporate subtype-specific assessment of public health significance in food control strategies and regulations.

## Conclusion and Future Perspectives

**FIGURE 3:**
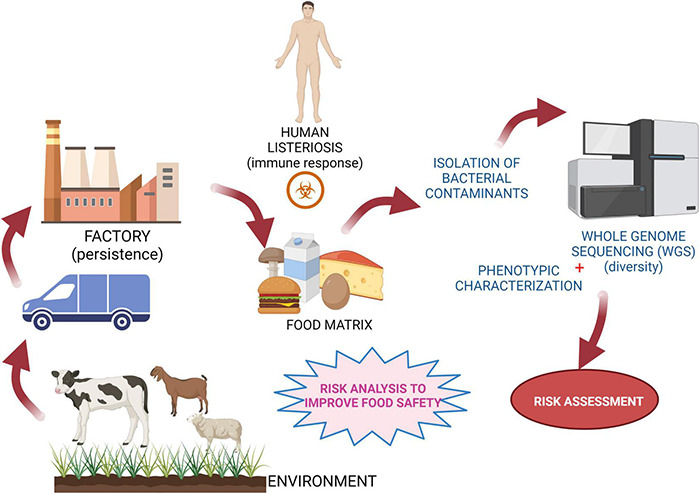
Schematic representation of risk control strategies. Experimental concept to assess risk and monitor diversity among *L. monocytogenes* strains for prevention/control of food chain contamination. Created with BioRender.com.

The notorious foodborne pathogen *L. monocytogenes* is ubiquitous in nature and can be found in soil, in the farm environment, in the food production environment, and in food products (Figure 3) (Kallipolitis et al., 2020). As highlighted in this review, there is a high degree of strain divergence regarding virulence potential, environmental adaption, and stress response. In addition, persistent *L. monocytogenes* subtypes have the ability to survive and persist for months and even years in food-processing environments and to keep contaminating food products. It is, however, difficult to correlate adaptive traits directly to persistence. Hence, pheno-genotype association studies are promising approaches to increase our mechanistic understanding of how this pathogen survives along the food chain and infects the human host. Notably, most published studies do not assess the presence/absence of specific genes (or sets of genes) in all currently known *L. monocytogenes* lineages, CCs/sequence types. Hence, experimental and holistic approaches based on WGS and environmental studies may play a role in determining the distribution and diversity of *Listeria* species. The use of advanced diagnostic technologies such as WGS can open avenues to fine-tune risk assessments, which is of great importance in the prevention and control of both animal and human listeriosis (Den Besten et al., 2018; Rantsiou et al., 2018). Successful implementation and use of WGS needs, however, an appropriate and functioning infrastructure and resources (Grace, 2015), i.e., functional control and an already established surveillance system to collect isolates and metadata from clinical, food, and environmental samples (EFSA, 2008; FAO, 2016). This will support an unbiased assessment of the likelihood of subtypes to be present in food and to cause disease to come to risk-based interventions at the intraspecific level.

## Author Contributions

All authors listed have made a substantial, direct, and intellectual contribution to the work, and approved it for publication.

## Conflict of Interest

The authors declare that the research was conducted in the absence of any commercial or financial relationships that could be construed as a potential conflict of interest.

## Publisher’s Note

All claims expressed in this article are solely those of the authors and do not necessarily represent those of their affiliated organizations, or those of the publisher, the editors and the reviewers. Any product that may be evaluated in this article, or claim that may be made by its manufacturer, is not guaranteed or endorsed by the publisher.

## Footnotes

1
http://bigsdb.pasteur.fr/listeria
